# Clinical Effects of Surgical Left Atrial Reduction and Concomitant Mitral Valve Replacement in Patients with Giant Left Atrium

**DOI:** 10.21470/1678-9741-2022-0469

**Published:** 2023-07-18

**Authors:** Song Yang, Cuiping Wang, Bao Zhang, Jian Hou, Suiqing Huang, Keke Wang, Zhongkai Wu

**Affiliations:** 1 Cardiothoracic Surgical Intensive Care Unit, the First Affiliated Hospital, Sun Yat-sen University, Guangdong, People’s Republic of China; 2 Cardiac Surgery Department, the First Affiliated Hospital, Sun Yat-sen University, Guangdong, People’s Republic of China; 3 Emergency Room, the First Affiliated Hospital, Sun Yat-sen University, Guangdong, People’s Republic of China

**Keywords:** Giant Left Atrium, Left Atrial Reduction, Mitral Valve Replacement

## Abstract

**Introduction:**

A giant left atrium may cause respiratory dysfunction and hemodynamic disturbance postoperatively. This retrospective study aimed to evaluate clinical effects of surgical left atrial reduction in concomitant cardiac valves operations.

**Methods:**

One hundred and thirty-five patients with heart valve diseases and giant left atriums from January 2004 to July 2021 were enrolled into this research. They were divided into the folded group (n=63) and the unfolded group (n=72). Patients in the folded group had undergone cardiac valve operations concomitantly with left atrial reductions. The perioperative characteristics were compared between both groups, and subgroup analysis was performed.

**Results:**

There were five deaths in the folded group and 25 deaths in the unfolded group (P<0.001). Complications including pneumonia, sepsis, multiple organs dysfunction syndrome, low cardiac output syndrome, and the use of continuous renal replacement therapy were significantly fewer in the folded group. The receiver operating characteristic curve of left atrial max. diameter predicting mortality was significant (area under the curve=0.878, P=0.005), and the cutoff point was 96.5 mm. The stratified analysis for sex showed that more female patients died in the unfolded group. Logistic regression for mortality showed that the left atrium unfolded, left atrial max. diameter, cardiopulmonary bypass time, and mechanical ventilation time increased the risk of death.

**Conclusion:**

Surgical left atrial reduction concomitantly with valves replacement could decrease mortality and was safe and effective in giant left atrium patients.

## INTRODUCTION

**Table:** 

Abbreviations, Acronyms & Symbols				
ACC	= Aortic cross-clamping		LA	= Left atrial
AF	= Atrial fibrillation		LCOS	= Low cardiac output syndrome
AKI	= Acute kidney injury		LVEF	= Left ventricular ejection fraction
AUC	= Area under the curve		MODS	= Multiple organs dysfunction syndrome
CAD	= Coronary artery disease		MVR	= Mitral valve replacements
CPB	= Cardiopulmonary bypass		MVT	= Mechanical ventilation time
CRRT	= Continuous renal replacement therapy		NS	= No significance
cTnT	= Cardiac troponin T		NT-proBNP	= N-terminal pro brain natriuretic peptide
DM	= Diabetes mellitus		NYHA	= New York Heart Association
DVR	= Double valve replacements		ROC	= Receiver operating characteristic
ECMO	= Extracorporeal membrane oxygenation		S	= Small
IABP	= Intra-aortic balloon pump		TVP	= Tricuspid valve plasty
ICU	= Intensive care unit		TVR	= Tricuspid valve replacements
L	= Large			

Left atrial (LA) enlargement is commonly witnessed with cardiac valve disease. Increased LA volume is associated with atrial fibrillation (AF) and the risk of thrombus formation^[1]^. Excessive LA enlargement can lead to bronchial compression or hoarseness of voice due to compression of the recurrent laryngeal nerve^[2]^. Giant left atrium is a rare condition defined by a LA diameter > 65 mm and is often associated with long-standing rheumatic mitral stenosis^[3]^. A giant left atrium may cause respiratory dysfunction and hemodynamic disturbance postoperatively. While operating for correction of mitral valve diseases, enlarged left atriums were reduced surgically^[4]^. Some surgical groups recommend routine LA reduction with only a modest increase in its size and without any clinical signs of LA enlargement^[5]^. There are no standard recommendations for this procedure based on the size of the LA^[4]^. Most surgeons fix the mitral valve and do little to an oversized left atrium. Others occlude the LA appendage^[6]^. We believe that a good proportion of surgeons think that successful mitral valve surgery alone will result in the eventual remodeling of the left atrium and size reduction. Risk of excessive bleeding, increased cardiopulmonary bypass (CPB) time, and unclear surgical efficacy raise many questions about the optimal approach to giant LA reduction^[7]^.

The purpose of this report is to assess the results of concomitant LA reduction with valve replacements in the treatment of heart valve diseases.

## METHODS

### Study Design and Patients

This was a retrospective single-center study. The inclusion criteria were adult patients older than 18 years who underwent valve replacements and the presence of giant left atriums. The exclusion criteria were a history of any mechanical assistance due to organ failure and usage of preoperative mechanical assistance. Overall, 135 patients (53 males and 82 females) in our center from January 2004 to July 2021 were enrolled into the study. All patients were divided into the folded group (n=63) and the unfolded group (n=72). The patients in the folded group had undergone traditional cardiac operations concomitantly with LA reductions, and in the unfolded group, LA reductions were not performed. There was stratified analysis for sex in both groups. Receiver operating characteristic (ROC) curves were plotted for factors that correlated significantly with LA max. diameter in the preoperative period and death in the folded group. Then a subgroup analysis was performed in the folded group according to the cutoff point of preoperative LA max. diameter. The logistic regression analysis was performed to determine predictive factors regarding death in all patients.

### Baseline Clinical Variables and Complications

Baseline clinical variables including age, gender, concomitant diseases, preoperative N-terminal pro brain natriuretic peptide, cardiac troponin T, serum creatine, procalcitonin, echocardiography-measured LA max. diameter, and left ventricular ejection fraction were compared. Complications included postoperative active bleeding, acute kidney injury (AKI), pneumonia, sepsis, multiple organs dysfunction syndrome (MODS), low cardiac output syndrome (LCOS), intra-aortic balloon pump, extracorporeal membrane oxygenation, and continuous renal replacement therapy (CRRT).

### Valve Replacement and Left Atrial Reduction Procedures

Standard CPB was established through median sternotomy. After the heart was stopped by aortic cross-clamping (ACC), we performed the mitral valve replacement (MVR) firstly. Then, we ligated the LA auricle or closed it by suturing inside the left atrium. LA reduction procedure was followed. We then performed the aortic valve replacement and tricuspid valve plasty (TVP), if needed. The patients were returned to the cardiac surgery intensive care unit (ICU) after the operations. Postoperative therapies were the same as those in conventional cardiac operations.

LA reduction procedure was performed according to the shape of the left atrium. Reduction techniques were made by application of LA free walls with double-row suture and without excision of atrial tissues. Three reduction technologies were used in the operations: ① plication of interatrial septum and sealing of LA appendage; ② para-annular plication of LA wall beside the mitral valve; ③ plication of the area between the right and the left pulmonary veins.

### Echocardiography

Echocardiography was performed routinely using Vivid™ Echocardiography System (GE, United States of America). Three LA diameters were recorded, including the anteroposterior diameter, superoinferior diameter, and left-right diameter. The max. diameter was chosen as the LA max. diameter.

### Statistical Analysis

All statistical analyses were performed using IBM Corp. Released 2015, IBM SPSS Statistics for Windows, version 23.0, Armonk, NY: IBM Corp. The results are expressed as mean values ± standard deviation or as numbers and percentages, as appropriate. Parametric and non-parametric tests were used for comparisons of continuous data appropriately. Chi-square test was used for comparisons of categorical data. ROC curves were plotted for factors that correlated significantly with LA max. diameter in the preoperative period and mortality. Univariate analyses were performed to determine predictive factors regarding death. All reported *P*-values were based on two-sided tests, and a *P*-value < 0.05 was considered significant.

## RESULTS

Baseline and perioperative characteristics between the folded group and the unfolded group were showed in Table 1. Procalcitonin in the folded group was lower than in the unfolded group (0.06±0.12 *vs.* 0.15±0.2, *P*=0.003). LA max. diameters had no significant difference between the folded group and the unfolded group (88.2±29.9 *vs.* 81.7±16.9, *P*=0.13). In the folded group, there were 41 MVR, 18 double valve replacements (DVR), and three tricuspid valve replacements (TVR). In the unfolded group, there were 54 MVR and 17 DVR. The concomitant procedures of TVP in the folded group were similar to those in the unfolded group (54 *vs.* 53, *P*=0.084). There were more complications in the unfolded group including pneumonia, sepsis, MODS, LCOS, and CRRT. The ICU stay time and mechanical ventilation time had no significant differences. There were five deaths in the folded group and 25 deaths in the unfolded group (*P*<0.001). The ROC curve of LA max. diameter predicting death was analyzed in the folded group (Figure 1). The area under the curve was 87.8%, and the *P*-value was 0.005. The cutoff point of LA max. diameter was 96.5 mm.

**Table 1:** Baseline and perioperative characteristics.

Characteristic	Folded group (n=63)	Unfolded group (n=72)	*P*-value
Age, years	51.3±10.1	54.3±12.6	0.137
Male, no.	26 (41.3%)	27 (37.5%)	0.655
AF, no.	50 (79.4%)	56 (77.8%)	0.823
Hypertension, no.	5 (7.9%)	9 (12.5%)	0.386
DM, no.	1 (1.6%)	5 (6.9%)	0.276
CAD, no.	4 (6.3%)	3 (4.2%)	0.856
Cerebral infarction, no.	9 (14.3%)	6 (8.3%)	0.272
NT-proBNP, pg/ml	1383.3±1263.7	1627.2±1750.4	0.361
cTnT, ug/L	0.04±0.09	0.05±0.09	0.614
Serum creatine, µmol/L	83.9±36.2	81.5±20.2	0.631
Procalcitonin, ng/ml	0.06±0.12	0.15±0.2	0.003
Left atrial max. diameter, mm	88.2±29.9	81.7±16.9	0.13
LVEF, %	61.9±8.8	61.2±9.1	0.673
NYHA, no.			0.828
II	15	18	
III	42	45	
IV	6	9	
Operation, no.			0.310
MVR	41	54	
DVR	19	17	
TVR	3	1	
Concomitant procedure, no.			
TVP	54 (85.7%)	53 (73.6%)	0.084
CPB, min.	162±61.2	183.3±117.2	0.182
ACC, min.	94.4±42.6	102.2±71.9	0.452
ICU stay time, days	3.7±5.4	4.1±4.4	0.582
MVT, hours	47.5±104.9	68.8±108.1	0.251
Postoperative bleeding, no.	0	3 (4.2%)	0.292
AKI, no.	18 (28.6%)	18 (25%)	0.64
Pneumonia, no.	14 (22.2%)	40 (55.6%)	< 0.001
Sepsis, no.	3 (4.8%)	19 (26.4%)	0.002
MODS, no.	5 (7.9%)	25 (34.7%)	< 0.001
LCOS, no.	11 (17.5%)	26 (36.1%)	0.015
IABP, no.	5 (7.9%)	12 (16.7%)	0.127
ECMO, no.	1 (1.6%)	4 (5.6%)	0.447
CRRT, no.	3 (4.8%)	13 (18.1%)	0.034
Death, no.	5 (7.9%)	25 (34.7%)	< 0.001

Data are expressed as mean ± standard deviation or as n (%) ACC=aortic cross-clamping; AF=atrial fibrillation; AKI=acute kidney injury; CAD=coronary artery disease; CPB=cardiopulmonary bypass; CRRT=continuous renal replacement therapy; cTnT=cardiac troponin T; DM=diabetes mellitus; DVR=double valve replacement; ECMO=extracorporeal membrane oxygenation; IABP=intra-aortic balloon pump; ICU=intensive care unit; LCOS=low cardiac output syndrome; LVEF=left ventricular ejection fraction; MODS=multiple organs dysfunction syndrome; MVR=mitral valve replacement; MVT=mechanical ventilation time; NT-proBNP=N-terminal pro brain natriuretic peptide; NYHA=New York Heart Association; TVP=tricuspid valve plasty; TVR=tricuspid valve replacement

**Fig. 2:**
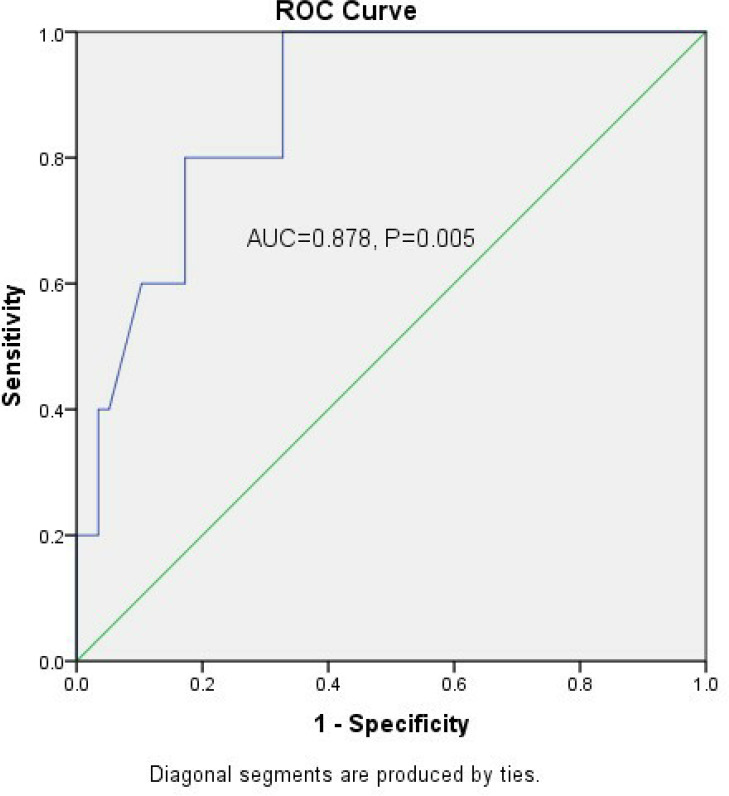
Receiver operating characteristic (ROC) curve. ROC curve for left atrial max. diameter predicting mortality. The cutoff point of left atrial max. diameter was 96.5 mm. AUC=area under the curve.

According to the cutoff point of LA max. diameter, a subgroup analysis was performed in the folded group. The 63 patients were divided into the small (S) group (LA max. ≤ 96.5 mm, n=39) and the large (L) group (LA max. > 96.5 mm, n=24). Baseline characteristics were showed in Table 2. LA max. diameters in the S group were significant smaller than in the L group (69.6±18.5 *vs.* 118.3±17.5, *P*<0.001). The CPB times were shorter in the S group than in the L group (*P*=0.04). But the ACC time had no significant difference. The ICU stay time in the S group was significantly shorter than in the L group (2.2±1.0 *vs.* 6.0±8.2, *P*=0.032). Mechanical ventilation time in the S group was 17.2±11.0 hours, while it was 96.8±159.2 hours in the L group (*P*=0.023). AKI and LCOS in the S group were fewer than in the L group (*P*<0.05). All the five deaths occurred in the L group (*P*=0.013).

**Table 2:** Subgroup analysis (S group: left atrial max. diameter < 96.5 mm; L group: left atrial max. diameter ≥ 96.5 mm).

Characteristic	S group (n=39)	L group (n=24)	*P*-value
Age, years	51.2±9.4	51.6±11.4	0.859
Male, no.	18 (46.2%)	8 (33.3%)	0.315
AF, no.	28 (71.8%)	22 (91.7%)	0.058
Hypertension, no.	3 (7.7%)	2 (8.3%)	1.0
DM, no.	1 (2.6%)	0	1.0
CAD, no.	4 (10.3%)	0	0.276
Cerebral infarction, no.	7 (17.9%)	2 (8.3%)	0.491
NT-proBNP, pg/ml	1173.6±1106.5	1724±1444.4	0.118
cTnT, ug/L	0.04±0.09	0.05±0.09	0.626
Serum creatine, µmol/L	86.1±41.3	80.4±26.3	0.553
Procalcitonin, ng/ml	0.06±0.12	0.07±0.11	0.602
Left atrial max. diameter, mm	69.6±18.5	118.3±17.5	< 0.001
LVEF, %	61.9±8.9	61.8±8.7	0.969
NYHA, no.			0.296
II	9	6	
III	28	14	
IV	2	4	
Operation, no.			0.295
MVR	28	13	
DVR	10	9	
TVR	1	2	
Concomitant procedure, no.			0.125
TVP	36 (92.3%)	18 (75%)	
CPB, min.	149.7±49	182.1±73.9	0.04
ACC, min.	89.6±31.2	102.1±56.4	0.328
ICU stay time, days	2.2±1.0	6.0±8.2	0.032
MVT, hours	17.2±11.0	96.8±159.2	0.023
Postoperative bleeding, no.	0	0	NS
AKI, no.	6 (15.4%)	12 (50%)	0.003
Pneumonia, no.	6 (15.4%)	8 (33.3%)	0.096
Sepsis, no.	0	3 (12.5%)	0.098
MODS, no.	1 (2.6%)	4 (16.7%)	0.126
LCOS, no.	2 (5.1%)	9 (37.5%)	0.003
IABP, no.	1 (2.6%)	4 (16.7%)	0.126
ECMO, no.	0	1 (4.2%)	0.805
CRRT, no.	0	3 (12.5%)	0.098
Death, no.	0	5 (20.8%)	0.013

Data are expressed as mean ± SD or as n (%). ACC=aortic cross-clamping; AF=atrial fibrillation; AKI=acute kidney injury; CAD=coronary artery disease; CPB=cardiopulmonary bypass; CRRT=continuous renal replacement therapy; cTnT=cardiac troponin T; DM=diabetes mellitus; DVR=double valve replacement; ECMO=extracorporeal membrane oxygenation; IABP=intra-aortic balloon pump; ICU=intensive care unit; L=large; LCOS=low cardiac output syndrome; LVEF=left ventricular ejection fraction; MODS=multiple organs dysfunction syndrome; MVR=mitral valve replacement; MVT=mechanical ventilation time; NS=no significance; NT-proBNP=N-terminal pro brain natriuretic peptide; NYHA=New York Heart Association; S=small; TVP=tricuspid valve plasty; TVR=tricuspid valve replacement

We performed a stratified analysis for sex in both groups, and the results were showed in Table 3. It was found that the serum creatine was higher in male patients than in female patients in both groups. LA diameters had no significant differences between the two groups. In the unfolded group, more patients died in the female subgroup than in the male subgroup. Other characteristics had no significant differences in the stratified analysis.

**Table 3:** Stratified analysis for sex in both groups.

Characteristic	Folded group (n=63)			Unfolded group (n=72)		
Subgroup	Female (n=37)	Male (n=26)	*P*-value	Female (n=45)	Male (n=27)	*P*-value
Age, years	50.0±9.3	53.2±11.1	0.187	54.7±12.2	53.6±13.4	0.658
AF, no.	30 (81.1%)	20 (76.9%)	0.688	37 (82.2%)	19 (70.4%)	0.242
Hypertension, no.	1 (2.7%)	4 (15.4%)	0.174	7 (15.6%)	2 (7.4%)	0.52
DM, no.	1 (2.7%)	0	1	4 (8.9%)	1 (3.7%)	0.72
CAD, no.	0	4 (15.4%)	0.052	2 (4.4%)	1 (3.7%)	1
Cerebral infarction, no.	5 (13.5%)	4 (15.4%)	1	3 (6.7%)	3 (11.1%)	0.826
NT-proBNP, pg/ml	1391.9±1246.3	1371.1±1312.8	0.802	1794.7±2078.4	1347.9±962.8	0.496
cTnT, ug/L	0.05±0.09	0.04±0.08	0.405	0.06±0.12	0.04±0.02	0.506
Serum creatine, µmol/L	74.6±21.4	97.2±47.8	0.003	76.2±20.5	90.4±16.3	0.001
Procalcitonin, ng/ml	0.06±0.13	0.06±0.1	0.644	0.12±0.09	0.2±0.3	0.308
Left atrial max. diameter, mm	86.6±28.3	90.3±32.5	0.743	81.3±13.3	82.2±22	0.345
LVEF, %	62±6.8	61.7±11.1	0.939	61.1±8.5	61.5±10.2	0.848
NYHA, no.			0.36			0.959
II	11	4		11	7	
III	22	20		28	17	
IV	4	2		6	3	
Operation, no.			0.959			0.594
MVR	24	17		34	20	
DVR	11	8		10	7	
TVR	2	7		1	0	
TVP, no.	33 (89.2%)	21 (80.8%)	0.566	33 (73.3%)	20 (74.1%)	0.945
CPB, min.	166.2±74.7	156.2±34.6	0.576	191±138.3	170.4±69.8	0.718
ACC, min.	94.5±51.1	94.2±27.1	0.534	107±85.3	94±41.1	0.776
ICU stay time, days	4.1±6.7	3±2.7	0.886	4.5±4.9	3.4±3.4	0.481
MVT, hours	62.9±133.4	25.7±28.2	0.463	79.7±118.1	50.5±88	0.264
Postoperative bleeding, no.	0	0	NS	2 (4.4%)	1 (3.7%)	1
AKI, no.	9 (24.3%)	9 (34.6%)	0.373	14 (31.1%)	4 (14.8)	0.206
Pneumonia, no.	5 (13.5%)	9 (34.6%)	0.094	28 (62.2%)	12 (44.4)	0.142
Sepsis, no.	1 (2.7%)	2 (7.7%)	0.753	14 (31.1%)	5 (18.5%)	0.241
MODS, no.	2 (5.4%)	3 (8.1%)	0.679	20 (44.4%)	5 (18.5%)	0.025
LCOS, no.	6 (16.2%)	5 (19.2%)	0.756	19 (42.2%)	7 (25.9%)	0.163
IABP, no.	2 (5.4%)	3 (8.1%)	0.679	10 (22.2%)	2 (7.4%)	0.191
ECMO, no.	0	1 (3.8%)	0.858	3 (6.7%)	1 (3.7%)	1
CRRT, no.	2 (5.4%)	1 (3.8%)	1	10 (22.2%)	3 (11.1%)	0.384
Death, no.	2 (5.4%)	3 (8.1%)	0.679	20 (44.4%)	5 (18.5%)	0.025

Data are expressed as mean ± standard deviation or as n (%). ACC=aortic cross-clamping; AF=atrial fibrillation; AKI=acute kidney injury; CAD=coronary artery disease; CPB=cardiopulmonary bypass; CRRT=continuous renal replacement therapy; cTnT=cardiac troponin T; DM=diabetes mellitus; DVR=double valve replacement; ECMO=extracorporeal membrane oxygenation; IABP=intra-aortic balloon pump; ICU=intensive care unit; LCOS=low cardiac output syndrome; LVEF=left ventricular ejection fraction; MODS=multiple organs dysfunction syndrome; MVR=mitral valve replacement; MVT=mechanical ventilation time; NS=no significance; NT-proBNP=N-terminal pro brain natriuretic peptide; NYHA=new York heart association; TVP=tricuspid valve plasty; TVR=tricuspid valve replacement

Logistic regression for death has been performed in all patients (Table 4). Left atrium unfolded, left atrial max. diameter, CPB time, and mechanical ventilation time were the risk factors of death. Therein, the odds ratio of the unfolded left atrium was 22.72.

**Table 4:** Results of logistic regression for death.

Variables	Odds ratio	95% confidence interval	*P*-value
Unfolded left atrium	22.72	2.916-176.956	0.003
Left atrial max. diameter	1.04	1.005-1.075	0.026
CPB	1.01	1.001-1.014	0.024
Mechanical ventilation time	1.02	1.007-1.024	< 0.001

CPB=cardiopulmonary bypass

## DISCUSSION

Our results demonstrated that LA reduction concomitant with valve replacement was safe and effective. The left atrium enlarged usually in rheumatic heart disease, especially in mitral valve disease, because of increased LA pressure and volume. The giant left atrium was known as the independent risk factor of AF. AF would decrease the cardiac output and cause atrial embolism^[8]^. The giant left atrium could compress the trachea, bronchus, left ventricle, lung, esophagus, and recurrent laryngeal nerve surrounding. At the same time, the giant left atrium meant a longer time of illness and serious valves diseases. It predicted bad outcomes or more complications^[4]^. Kawazoe^[9]^ reported that among patients who underwent valve operation but a giant left atrium was left free, 70% presented LCOS, 40% respiratory failure, and 40% died. These complications and mortality would decrease if valve operation was combined with LA reduction. Therefore, positive LA reduction was suggested in the course of valve replacements or plasty. In the present study, there were 25 deaths in the unfolded group and five deaths in the folded group. Pneumonia, sepsis, MODS, LCOS, and CRRT were significantly fewer in the folded group than in the unfolded group. These results were similar to other centers’ results^[5,7,10-15]^. It might suggest that LA reduction could decrease mortality and incidence of complications.

There were several methods of plication^[4,14,16-18]^. The left atrium might enlarge forward different directions. Different effects resulted from performing different atrial plication parts. For example, para-annular plication of LA wall among the mitral valve could mitigate the compression to the left ventricle and esophagus. Plication of interatrial septum could mitigate the compression to the right atrium. Therefore, according to the shape of the left atrium, we have chosen different kinds of plication to reduce LA volume. We suggest that the plication should be performed from inside of the left atrium. A suture from outside the left atrium might affect the pulmonary veins reflux. At the same time, it might easily cause atrial embolism for the rough intima. Some surgeons performed the left atrium plications when the heart was beating from outside the left atrium. We personally thought that was not suitable because it could result in more complications and bad clinical outcomes such as postoperative bleeding and mortality. Nevertheless, cardiac autotransplantation^[19,20]^ was thought to be the most radical technique of giant LA reduction which allowed maximally reducing all parts including interatrial septum. But the relative complexity and prolonged operative time made cardiac autotransplantation to be a technically demanding procedure and recommended only in case with extreme range of LA enlargement^[1]^. Therefore, there wasn’t a sole standard plication method for giant left atrium. We could choose suitable plication methods according to the geometry of the giant left atrium.

AF was often concomitant in patients who had mitral valve disease and enlarged left atrium. An enlarged left atrium was a well-known risk factor for ablation failure of AF. Kasemsarn^[7]^ had performed mitral valve surgery concomitant with AF radiofrequency ablation and found that the preoperative LA diameter > 50 mm was the predictor of recurrence of AF. Kim^[10]^ had found that surgical AF ablation without LA volume reduction was inappropriate for patients with a giant left atrium and AF. Sunderland^[21]^ suggested that patients with an enlarged (≥ 55 mm) LA who were at risk of failing to obtain sinus conversion after a standard maze procedure might derive benefit from concomitant atrial reduction surgery using either a tissue excision or a tissue plication technique. In the present study, we did not perform AF radiofrequency ablation because of the giant left atriums and the patients’ willingness.

Giant left atrium was the end product of severe and prolonged pressure and volume overload, occurring mainly during mitral insufficiency, stenosis, and, rarely, in mitral valve prolapse alone^[4]^. With the time going on, the left atrium could become larger and larger. Progressive LA dilatation might reflect the severity and duration of mitral valve disease^[22]^. At the same time, some remodeling happened on the LA wall^[23]^. Therefore, LA volume might be one of the risk factors for mitral valve disease. We have made the ROC curve for the LA max. diameter predicting death and found that the cutoff point of LA max. diameter was 96.5 mm (Figure 1). The patients of the folded group were divided into two subgroups according to the cutoff point. It was found that the L group had more complications and mortality than the S group. It meant that the enlargement of left atrium could predict the prognosis of patients, even though the LA reduction had been done. According to the results of logistic regression, there were four risk factors of death. The odds ratio of unfolded LA was 22.72. Therefore, the LA reduction procedure in such patients was strongly recommended.

### Limitations

There were some limitations in our study. The first one is that this was a single-center retrospective research. Secondly, the temporal distribution of patients was not balanced. We would try to do more works to perfect this question in the future.

## CONCLUSION

Surgical LA reduction concomitantly with valve replacements could decrease mortality and were safe and effective in giant left atrium patients.
